# Pharmacokinetic and pharmacodynamic analysis of 5-aza-2’-deoxycytidine (decitabine) in the design of its dose-schedule for cancer therapy

**DOI:** 10.1186/1868-7083-5-3

**Published:** 2013-02-01

**Authors:** Metin Karahoca, Richard L Momparler

**Affiliations:** 1 Département de Pharmacologie, Université de Montréal, Montréal, Québec, Canada; 2 Service of Hematology and Oncology, Centre de recherche, CHU-Saint-Justine, 3175 Côte Sainte-Catherine, Montréal, Québec, H3T 1C5, Canada

**Keywords:** Decitabine, 5-aza-2’-deoxycytidine, Epigenetics, AML, MDS, DNA methylation, Pharmacokinetics, Pharmacodynamics

## Abstract

5-Aza-2′-deoxycytidine (5-AZA-CdR, decitabine), an epigenetic drug that inhibits DNA methylation, is currently used to treat myelodysplastic syndrome (MDS), and is under investigation for treating acute myeloid leukemia (AML) and other malignancies. 5-AZA-CdR can reactivate tumor suppressor genes silenced by aberrant DNA methylation, a frequent event in all types of cancer. Because this epigenetic change is reversible, it is a good target for 5-AZA-CdR therapy. We have reviewed the preclinical data of 5-AZA-CdR to analyze the concentrations and exposure times required to eradicate cancer stem cells. We analyzed the dose-schedules used in animal models that show potent antineoplastic activity of 5-AZA-CdR. We attempted to correlate the preclinical data with the responses obtained in clinical trials of 5-AZA-CdR in patients with cancer. The pharmacokinetics and drug distribution of 5-AZA-CdR are key parameters because adequate therapeutic drug levels are required to eliminate cancer stem cells in all anatomic compartments. The plasma half-life of 5-AZA-CdR in humans is approximately 20 minutes due to the high levels in the liver of cytidine deaminase, the enzyme that inactivates this analogue. This provides a rationale to use an inhibitor of cytidine deaminase in combination with 5-AZA-CdR. Low-dose 5-AZA-CdR is effective for MDS and AML and can induce complete remissions (CR). However, maintenance of CR with low-dose 5-AZA-CdR is difficult. Based on analyses of preclinical and clinical data, low dose 5-AZA-CdR has the potential to be an effective form of therapy in some patients with cancer. For patients who do not respond to low dose therapy we recommend dose-intensive treatment with 5-AZA-CdR. Patients who are candidates for intensive dose 5-AZA-CdR should have a good bone marrow status so as to permit adequate recovery from myelosuppression, the major toxicity of 5-AZA-CdR. Solid tumors are also interesting targets for therapy with 5-AZA-CdR. Both low dose and intensive therapy with 5-AZA-CdR can reduce the proliferative potential of tumor stem cells in animal models. We propose novel dose schedules of 5-AZA-CdR for investigation in patients with cancer. The full chemotherapeutic potential of 5-AZA-CdR to treat cancer merits further clinical investigation and can only be realized when its optimal dose-schedule is determined.

## Introduction

5-Aza-2′-deoxycytidine (5-AZA-CdR, decitabine) was first synthesized in 1964 by Pliml and Sorm [1] and its potential activity in leukemia was reported in 1968 by Sorm and Vesely [2]. 5-AZA-CdR is an analog of the natural nucleoside 2′-deoxycytidine in which the carbon in the 5-position of the cytosine is replaced by nitrogen. Preclinical studies in rodents indicated that 5-AZA-CdR is a more potent antileukemic agent than cytosine arabinoside (ARA-C) [3,4]. In 1979, Taylor and Jones [5] reported that 5-AZA-CdR could induce cells in culture to differentiate into different phenotypes and this activity correlated with its inhibition of DNA methylation. The first review on the pharmacologic properties of 5-AZA-CdR was published in 1979 [6] and the first clinical trial on 5-AZA-CdR in patients with acute leukemia was published in 1981 [7].

Although the unique demethylating capacity of 5-AZA-CdR has been known for many years, its approval for the treatment of cancer took a long time, perhaps due to a lack of understanding of the importance of epigenetic changes in malignancy during the early years of development [8]. 5-AZA-CdR was approved for the treatment of myelodysplastic syndrome (MDS) in 2006 and shows anti-leukemic activity against acute myeloid leukemia (AML) as well [8,9]. Its clinical activity against solid tumors is under investigation. 5-AZA-CdR also displays some effectiveness in the treatment of sickle cell disease, which is nonmalignant [10]. Low-dose 5-AZA-CdR was proposed to be effective for treating MDS due to its epigenetic action, whereas higher doses were too toxic due the poor hematologic status of these patients [11]. The objectives of this review is to analyze preclinical and clinical data on 5-AZA-CdR and develop a rationale for optimization of its dose schedule for the treatment of cancer.

## Review

### Mode of action and cellular metabolism

Recent progress on elucidating the important role of epigenetics in the development of malignancy has generated more interest in the chemotherapeutic potential of 5-AZA-CdR for the treatment of cancer [12,13]. ‘Epigenetics’ refers to the study of heritable changes in gene expression that occur without changes in the DNA sequences [14]. Epigenetic regulation of gene expression is modulated by changes in DNA methylation, covalent histone modifications, and microRNA [15-17]. Hypermethylated tumor suppressor genes represent one of the most consistent hallmarks of human cancers [12,18]. Loss of the expression of tumor suppressor genes can lead to a loss of regulation of cancer cell growth [19-21].

DNA methylation usually occurs at the 5-position of the cytosine ring within cytosine-phosphate-guanine (CpG) dinucleotide by a transfer of the methyl group from *S*-adenosyl-l-methionine [22]. DNA methyltransferases (DNMTs) catalyze this reaction. The methylation pattern from the parental DNA is copied onto the newly synthesized DNA strand by the maintenance methyltransferase DNMT 1. In embryonic stem cells and tumor cells, methylation of previously unmethylated DNA (*de novo* methylation) is catalyzed by DNMT 3a or DNMT 3b [23]. To inactivate transcription, methylation usually occurs in the CpG islands in the promoter-exon regions of target genes. Half of all genes harbor CpG islands in their promoters [24]. In human DNA, approximately 50% to 70% of CpG dinucleotides are methylated [25,26]. During normal embryonic development, cytosine methylation is essential for establishing tissue-specific gene expression, silencing imprinted genes, and inactivating the X chromosome. Methylation also protects against the transcription of parasitic elements [27].

Both 5-AZA-CdR and 5-azacytidine have been reported to inhibit the expression of the *de novo* DNA methylating enzymes, DNMT3B [28,29]. DNMT3A-DNMT3B double null embryonic stem cells are highly resistant to 5-AZA-CdR as compared to single null or wild type cells [30]. Mutations in DNMT3A have been identified in AML and MDS [31,32]. These DNMT3A mutations are associated with a poor outcome for both AML and MDS patients. A recent report showed that DNMT3A plays a role in silencing self-renewal genes in hematopoietic stem cells so as to permit hematopoietic differentiation [33]. Preliminary data indicate that AML patients with low DNMT3A activity may benefit from treatment with 5-AZA-CdR [34]. The full role of DNMT3A and DNMT3B in leukemogenesis still remains to be clarified.

**Figure 1:**
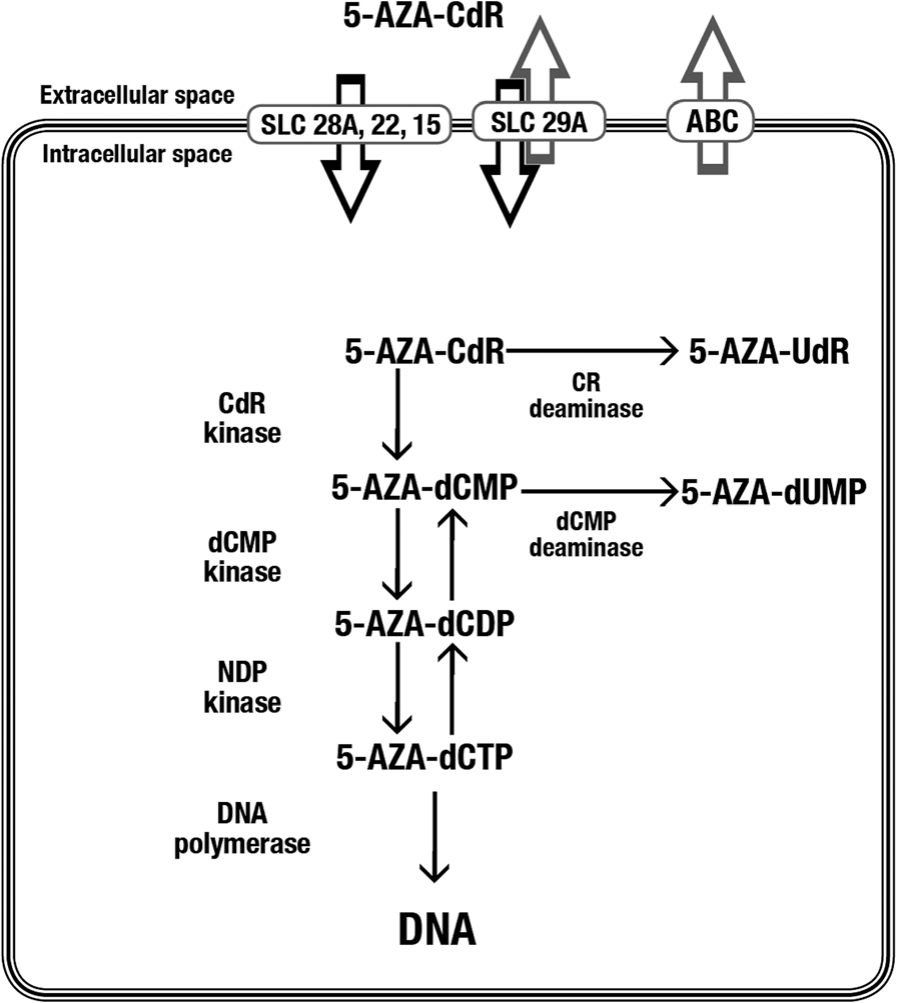
**Intracellular metabolism of 5-AZA-CdR.** 5-AZA-CdR is transported into the cell by the equilibrative-nucleoside transport system. 5-AZA-CdR converts into its triphosphate form by phosphorylation and binds covalently to the DNA, where it blocks DNMTs and causes demethylation of DNA. 5-AZA-CdR, 5-aza-2′-deoxycytidine; DNMTs, DNA methyltransferases.

5-AZA-CdR is a prodrug that must be activated by phosphorylation. The metabolism of this analog is summarized in Figure 1. Due to the function of the nucleoside transport system, 5-AZA-CdR rapidly reaches its equilibration state between the extracellular and intracellular compartments, as indicated by the short alpha half-life of five minutes [35].

The cellular uptake of the drug is realized by an equilibrative nucleoside-specific transport mechanism [36-39], which is followed by phosphorylation and incorporation of the drug into DNA, resulting in potent inhibition of DNMT.

Since 5-AZA-CdR is a prodrug it must be activated by deoxycytidine kinase to its monophosphate form and by other kinases to its triphosphate form, which is then incorporated into DNA by DNA polymerase [5,40-43]. The inhibition of DNA methylation is due to formation of a covalent complex between 5-AZA-CdR-DNA and DNMT1 at CpG methylation sites, resulting in the inactivation of this enzyme [44,45]. DNMT1 inhibition results in DNA hypomethylation, gene activation and the induction of cellular differentiation, senescence, and/or apoptosis [40-42].

5-AZA-CdR does not block the progression of G1-phase cells into S-phase [46]. Flow cytometry investigations revealed that 5-AZA-CdR slows the progression of cells into S-phase, but it does not block cell progression through their cycle [47], unlike the related nucleoside analogs 5-azacytidine and ARA-C [48]. One of the key biochemical markers of 5-AZA-CdR is the p15 tumor suppressor gene, which is frequently hypermethylated in MDS and AML patients and can be reactivated by this analog [49]. Table 1 summarizes key genes that are silenced by DNA methylation in different types of cancer as published in the review by Esteller [15].

**Table 1:** **Silencing by DNA methylation of key genes in different types of cancer**^**a**^

**Genes**	**Type of cancer**
p16, p14, RARβ2, SFRP1	Colon cancer
p16, p14	Esophageal cancer
p14, hMLH1	Stomach cancer
SOCS1, GSTP1	Liver cancer
ER, BRCA1, E-cadherin, TMS1	Breast cancer
BRCA1	Ovarian cancer
GSTP1	Prostate cancer
p16, DAPK, RASSFIA	Lung cancer
p16, TPEF/HPP1, miR-127	Bladder cancer
VHL	Kidney cancer
p15, EST1, ID4	Leukemia
p16, p73, MGMT	Lymphoma

^a^modified from Esteller M. [15]. p16, p16^INK4a^; p14, p14^ARF^; p15, p15^INK4b^.

## *In vitro* antineoplastic action

### Leukemia

*In vitro* studies of 5-AZA-CdR demonstrate that it induces differentiation, apoptosis, and senescence in leukemic cells [40-42,50-52]. One proposed mechanism of this action is that 5-AZA-CdR exposure reduces the expression of c-myc, an oncogene that prevents the terminal differentiation of cells [53]. 5-AZA-CdR also maintains hematopoietic precursor self-renewal [54]. Leukemic cells can undergo phenotypic modification after exposure to 5-AZA-CdR, as indicated by the increased expression of several antigen markers [55,56]. 5-AZA-CdR enhances the graft-versus leukemia effect, which has the potential to increase the immunologic therapeutic efficacy of allogeneic transplantation [57]. 5-AZA-CdR also induces the upregulation of DNA repair genes and angiogenesis inhibitor genes [58]. These changes can suppress the development of malignancy. *In vitro* studies of 5-AZA-CdR also illustrate its potent antileukemic activity [59,60]. At equimolar concentrations, 5-AZA-CdR more potently reduces colony formation than either 5-azacytidine or ARA-C [61]. 5-AZA-CdR also has greater DNA-hypomethylating activity than 5-azacytidine [61]. *In vitro* studies on the induction of the loss of clonogenicity by 5-AZA-CdR on human leukemic cell lines of different phenotypes are summarized in Table 2. The 50% inhibitory concentration (IC50) of colony formation for a one-hour exposure to 5-AZA-CdR was approximately 10 μM for HL-60 (myeloid), Molt-3 (T-cell) and RPMI-8392 (B-cell) leukemic cell lines. For a 24-hour exposure, the IC50 of 5-AZA-CdR is in the range of 0.1 μM for these leukemic cell lines. This observation illustrates the importance of the duration of treatment with respect to the antileukemic activity of 5-AZA-CdR.

**Table 2:** ***In vitro *concentrations of 5-AZA-CdR that eradicate the proliferative potential of leukemic cells**

**Leukemic cell lines**	**IC50**	**Duration of exposure**	**References**
HL 60 (myeloid)	100 μM	1 hour	[59]
Molt-3 (T-cell)	10 μM	1 hour	[59]
RPMI 8392 (B-cell)	10 μM	1 hour	[59]
HL60 leukemic cells	0.05 to 0.1 μM	24 hours	[60]
L1210 leukemic cells	0.075 μM	18 hours	[60]
Molt-3 (T-cell)	about 0.1 μM	24 hours	[59]
HL 60 (myeloid)	about 0.1 μM	24 hours	[59]
RPMI 8392 (B-cell)	about 0.1 μM	24 hours	[59]

1 μM = 228 ng/ml. 5-AZA-CdR, 5-Aza-2′-deoxycytidine; IC50, half maximal inhibitory concentration.

### In solid tumors

Several studies on different type of tumors demonstrated an association between DNA hypermethylation and DNMT overexpression [62-64]. The precise molecular mechanism of aberrant DNA methylation, however, remains to be clarified. The first tumor suppressor gene found to undergo silencing as a result of promoter methylation was the Rb gene in retinoblastoma tumors, followed by numerous other tumor suppressor genes [65-67]. 5-AZA-CdR reactivates many genes that suppress malignancy and are silenced by aberrant DNA methylation in solid tumors (Table 1). 5-AZA-CdR reactivates the tumor suppressor gene VHL in human renal carcinoma cell lines and the expression of the p16/CDKN2 tumor suppressor gene, which prevents the entry of tumor cells into the S-phase of the cell cycle, in different tumor cell lines [20,68-72]. The mRNA expression of the p53 gene increases in hepatoma cell lines after treatment with 5-AZA-CdR [73]. Methylation of hMLH1, a mismatch repair gene that confers chemoresistance to certain anticancer agents, is reactivated by 5-AZA-CdR in ovarian and colon cancer cell lines [74,75]. 5-AZA-CdR reactivates CDKN2a (p16INK4a) in lung cancer, BRCA1 in breast cancer, and MGMT in glioblastomas [76-79]. Expression of unmethylated genes, such as Apaf-1 and CDKN2D in malignant melanoma and lung cancer, is enhanced after 5-AZA-CdR treatment, indicating that gene activation can occur by indirect mechanisms [80,81]. 5-AZA-CdR reduces natural killer cell responsiveness, which may be used in therapeutic strategies to target malignant cells by an immune mechanism [82].

Preclinical studies using colony assays indicate that 5-AZA-CdR is an active antineoplastic agent against many different tumor cell lines [60,83-88]. These studies demonstrated that the demethylation of genes involved in cell cycle control inhibits the growth of various tumors [66,79,89]. The *in vitro* studies on the antineoplastic activity of 5-AZA-CdR in tumor cell lines are summarized in Table 3. For example, the IC50 is approximately 4 μM for a two-hour exposure in fibrosarcoma cells and approximately 0.5 μM for a four-hour exposure in Calu-6 lung carcinoma cells [60,88]. For a 48-hour exposure, the IC50 is in the range of 0.1 μM for different tumor cell lines (Table 3). As observed with leukemic cells, the duration of treatment is a key factor with respect to the antineoplastic activity of 5-AZA-CdR.

**Table 3:** ***In-vitro *concentrations of 5-AZA-CdR that eradicate proliferative potential of solid tumor cells**

**Tumor cell lines**	**IC 50**	**Duration of treatment**	**References**
HS-SY-II synovial sarcoma	1.3 μM	96 hours	[84]
SYO-1 synovial sarcoma	0.9 μM	96 hours	[84]
KB oropharyngeal cancer	0.5 μM	96 hours	[85]
A549 lung adenocancer	0.49 μM	96 hours	[85]
LoVo colon cancer	0.4 μM	96 hours	[85]
LoVo-DX colon cancer	0.1 μM	96 hours	[85]
MDA-MB- 435 breast cancer	0.2 μM	48 hours	[83]
Hs578T breast cancer	0.13 μM	48 hours	[87]
MCF-7 breast cancer	0.13 μM	48 hours	[87]
MDA-MB- 231 breast cancer	0.013 μM	48 hours	[83]
EMT6 mammary cancer	0.22 μM	18 hours	[60]
Calu-6 lung cancer	0.44 μM	4-8 hours	[60]
A(T1)C1-3 fibrosarcoma	4.38 μM	2 hours	[88]

1 μM= 228 ng/ml. 5-AZA-CdR, 5-Aza-2′-deoxycytidine; IC50, half maximal inhibitory concentration.

### *In vivo* antineoplastic action

The antineoplastic activity of 5-AZA-CdR was first demonstrated in mouse models of acute leukemia [2]. In mice with murine L1210 leukemia, the activity of 5-AZA-CDR markedly increases with an increased dose and exposure time [60,61]. In a preclinical study using the L1210 mouse leukemia model, the curative dose of 5-AZA-CdR administered as a 15-hour infusion was 20 mg/kg. The antileukemic activity of 5-AZA-CdR, 5-azacytidine, and ARA-C was compared in the L1210 leukemia mouse model [61]. The mice were administered these nucleoside analogs in a 15-hour continuous intravenous (i.v.) infusion to obtain an effective plasma concentration of the drug that persisted longer than the cell cycle of these leukemia cells (12 hours). At equitoxic doses, 5-azacytidine (11.7 mg/kg) increased the life span of the mice by 63%, whereas 5-AZA-CdR (10.1 mg/kg) increased the life span of the mice by 384%. At higher doses, 5-AZA-CdR cured the mice of leukemia, but 5-azacytidine or ARA-C did not. Because the cell cycle of L1210 leukemic cells is around 12 hours in duration, the 15-hour infusion of these analogs was predicted to produce 100% cell kill in this mouse model because all of the leukemic cells entered S-phase during the treatment. Analysis of the surviving leukemic cells revealed that they were not resistant to ARA-C.

In a rat model of myeloid leukemia, 5-AZA-CdR increased survival in a dose-related manner [4]. In this model, the antileukemic action of 5-AZA-CdR exceeded that of ARA-C. The hematologic toxicity of 5-AZA-CdR was evaluated in this rat model by the colony forming unit in the spleen (CFU-S) assay [4]. The maximum number of hematopoietic stem cells killed at high doses of 5-AZA-CdR was the same as that at low doses, indicating that the response reaches a plateau upon dose escalation. This is solid evidence that normal resting hematopoietic stem cells survive high-dose treatment with 5-AZA-CdR. The difference between hematopoietic stem cell toxicity and antileukemic effects favors treatment with high doses of 5-AZA-CdR because the dose that causes maximal leukemic cell kill is not more toxic to resting hematopoietic stem cells.

5-AZA-CdR displays significant antineoplastic activity against tumors in a mouse model. In mice bearing EMT6 mammary tumors, 5-AZA-CdR at a dose of 15 mg/kg produces a 2-log reduction in clonogenic survival as measured by excision of the tumor, preparation of a single-cell suspension and analysis of survival by *in vitro* colony formation [60]. A higher dose of 30 mg/kg produces a 3-log reduction in clonogenic survival. These observations illustrate the effectiveness of intensive doses of 5-AZA-CdR for tumor therapy.

## Pharmacokinetics of 5-AZA-CdR

### Methods used to determine concentrations of 5-Aza-CdR

Several methods are used to determine 5-AZA-CdR concentrations, including bioassays, HPLC, and HPLC combined with mass spectrometry (HPLC/MS). Among these methods, HPLC/MS is the most sensitive followed by bioassays; both of these methods are superior to HPLC alone for detecting low concentrations of 5-AZA-CdR. The lower limit of detection of 5-AZA-CdR is in the range of 0.01 μM. For pharmacokinetic studies, HPLC/MS is the method of choice for detecting the plasma concentration of 5-AZA-CdR followed by the bioassay and HPLC methods (Table 4) [7,36,90-92].

**Table 4:** Comparison of methods to determine concentration of 5-AZA-CdR

**Method**	**Limit sensitivity for detection**	**References**
Bioassay	5 nM	[7]
	6 nM	[36]
HPLC	750 nM	[90]
HPLC/MS	10 nM	[91]
	5 nM	[92]

1 μM = 228 ng/ml. 5-AZA-CdR, 5-Aza-2′-deoxycytidine; HPLC, high performance liquid chromatography; HPLC/MS, Hjgh performance liquid chromatography/mass spectrometry.

### Pharmacokinetic data

The moderate chemical instability of 5-AZA-CdR is important and dependent on both temperature and pH [93]. Therefore, this agent must be carefully formulated. The instability is due to the opening of the 5-azacytosine ring between positions 5 and 6 to form *N*-(formylamidino)-*N*′-β-d-2-deoxyribofuranosylurea (NFDU). This reaction is highly reversible in favor of 5-AZA-CdR and results in a minimal loss of pharmacologic activity. When NFDU decomposes irreversibly via loss of the formyl moiety to form *N*′-β-d-2-deoxyribofuranosyl-3-guanylurea (DGU), there is a complete loss of pharmacologic activity. This reaction occurs rapidly in alkaline solutions and increases with elevation of the temperature. At 24°C, the 50% decomposition rates (D50s) of 5-AZA-CdR to DGU at pH 7.0 and 8.5 are 22 hours and 5 hours, respectively. At 37°C, the D50s at pH 7.0 and 8.5 are 12hours and 2 hours, respectively. At 5°C and pH 7.0 to 7.4, the decomposition of 5-AZA-CdR to DGU is minimal (<1%). To formulate 5-AZA-CdR, we recommend using 5 to10 mM potassium phosphate, pH 7.0 to 7.4 and storage at 5°C until clinical use. A fresh solution of 5-AZA-CdR can be prepared every eight hours. During the infusion of 5-AZA-CdR to patients, we recommend using an ice pack to maintain the temperature at 10°C [93]. After i.v. administration of 5-AZA-CdR, its binding to plasma protein is less than 1%. There is a rapid distribution of 5-AZA-CdR between the extra- and intracellular compartments after i.v. injection, as indicated by its alpha half-life of five minutes [35].

**Figure 2:**
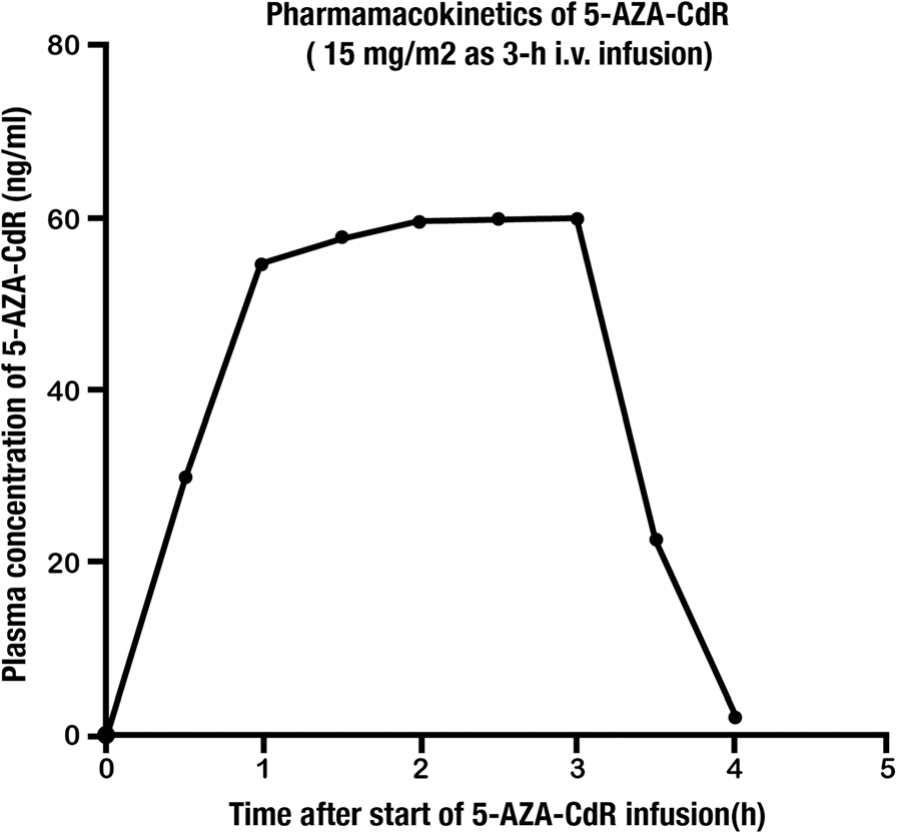
**Pharmacokinetics of 5-AZA-CdR at a dose of 15 mg/m**^**2 **^**as a three-hour i.v. infusion to patients with AML and MDS [**[92]**].** After i.v. infusion of 5-AZA-CdR, it reaches a steady-state plasma level within two hours, confirming its very short half-life. 5-AZA-CdR, 5-aza-2′-deoxycytidine; AML, acute myeloid leukemia; i.v., intravenous; MDS, myelodysplastic syndrome.

5-AZA-CdR penetrates the blood–brain barrier. The level of 5-AZA-CdR in the cerebrospinal fluid (CSF) reaches as high as 50% of its plasma level during a continuous infusion [35]. The beta plasma half-life of 5-AZA-CdR in adults is approximately 15 to 25 minutes due to very high levels of cytidine deaminase in the human liver and spleen [94,95] In pediatric populations, the beta half-life is 10 to 15 minutes, which is shorter than that of adults, probably due to the higher cytidine deaminase activity in the liver and spleen of children [7,90]. Repeated administration of 5-AZA-CdR in patients with MDS as a 3-hour infusion of 15 mg/m^2^ every eight hours for three days does not result in systemic accumulation of the drug, and the pharmacokinetic parameters remain unchanged from cycle to cycle [92]. An illustration of the plasma pharmacokinetics of the dose schedule that is currently used to treat MDS and AML is shown in Figure 2. 5-AZA-CdR is cleared from the systemic circulation rapidly. 5-AZA-CdR is inactivated primarily through deamination by cytidine deaminase in the human liver and spleen [94,95]. The half-life of 5-AZA-CdR is related to the blood flow into the liver. Its clearance exceeds the total renal capacity, which suggests an important role of non-renal elimination. The clearance rate has been reported as approximately 125 ± 20 ml/min/kg [36]. The urinary excretion of unchanged 5-AZA-CdR is low, typically <1% of the total dose given to the patients [36]. Oral formulations of 5-AZA-CdR are currently under investigation [96]. The pharmacokinetic parameters of 5-AZA-CdR are summarized in Table 5.

**Table 5:** Clinical studies on pharmacokinetics of 5-Aza-CdR

**Method (reference)**	**Dose-schedule**	**Plasma β half-life (minutes)**	**Clearance(ml/min/kg)**
Bioassay[36]	25 to 100 mg/m^2^ 1 hour inf q8 hours	35 +/− 5	126 +/− 21
LCMS[92]	15 mg/m^2^ 3hour inf q8 hours	35	127-135
HPLC[90]	30 mg/m^2^ 40 to 60 hours inf	10 to 15	-

5-AZA-CdR, 5-aza-2-deoxycytidine; inf, infusion; LCMS, liquid chromatography/mass spectrometry.

### 5-Aza-CdR in the treatment of hematologic malignancies

5-AZA-CdR was first used as a single agent in a phase I study in children with relapsed or refractory acute leukemia [7]. Escalating doses of 5-AZA-CdR between 0.75 and 80 mg/kg were explored. When 5-AZA-CdR was given at doses of 1 to 25 mg/m^2^/hour for 12 to 30 hours, only minor responses were observed. At a dose of 25 to 50 mg/m^2^/hour for 36 to 44 hours (2.35 to 4.70 μM), two complete remissions (CRs) were observed among nine patients. The authors reported a significant reduction of circulating blasts at all dose levels. In addition, 5-AZA-CdR cleared the CSF of two acute lymphocytic leukemia (ALL) patients with central nervous system involvement. No maximum tolerated dose (MTD) was reported. The major toxicity was myelosuppression that occurred at doses of 36 to 80 mg/kg infused over 36 to 44 hours. In a continuation of this study, 27 pediatric acute leukemia patients were treated with a continuous infusion of 5-AZA-CdR at doses ranging from 37 to 80 mg/kg over 36 to 60 hours. The predicted steady-state plasma level of 5-AZA-CdR for this schedule is 1.5 to 5 μM. The overall objective response rate was 37% (33% in ALL patients and 50% in AML patients) [97]. In this phase I trial, the MTD was estimated to be in the range of 1,500 to 2,250 mg/m^2^[7,97]. Despite promising outcomes, the development of the drug was discontinued due to the risk of prolonged myelosuppression in patients with advanced leukemia [98].

Several clinical trials investigating different drug dosing schedules of 5-AZA-CdR demonstrated significant clinical benefits in the treatment of patients with MDS and AML [11,56,98-100]. 5-AZA-CdR was initially investigated in a trial of 10 MDS patients [101]. Patients were treated with 5-AZA-CdR at a daily dose of 45 mg/m^2^ divided into three four-hour infusions for three days (six patients) or as a continuous infusion of 50 mg/m^2^ for three days (four patients). 5-AZA-CdR induced an overall response rate of 50% with a complete hematologic response in 40% of patients. A subsequent clinical trial reported similar response rates [102]. The finding that low doses of 5-AZA-CdR (3 mg/kg/cycle) showed activity in patients with sickle cell anemia, by re-expressing the fetal globin gene [10], led to the suggestion that low-dose use of 5-AZA-CdR could be effective against this disease, as well as MDS [18,103]. This hypothesis was evaluated in a phase I study with various low-dose levels of 5-AZA-CdR in patients with MDS or leukemia [11]. Unlike traditional phase I studies, the goal of this study was not to determine the MTD, but to determine the optimal biologic dose of this drug based on the response and other related parameters. Forty-eight patients were enrolled, the majority with AML. Responses were observed at the intermediate dose level of 15 mg/m^2^ daily × 10 days (150 mg/m^2^/course). As a result, this dose level was investigated in 11 additional patients. Of the nine responders, eight achieved CR after one cycle of 5-AZA-CdR. Hematologic toxicities were common but difficult to distinguish from the underlying disease. Non-hematologic side effects were uncommon. Pharmacodynamic studies revealed that dose-dependent decreases in DNA methylation reached a plateau at approximately 150 to 200 mg/m^2^/course [104]. Hypomethylation was observed for the p15 tumor-suppressor gene, but there was no clear correlation between hypomethylation of this gene and the overall response [104]. These results suggested that response to 5-AZA-CdR can occur at levels below the MTD and that prolonged exposure increases the response.

5-AZA-CdR was approved by the Food and Drug Administration (FDA) in 2006 for the treatment of MDS on the basis of a phase III multicenter trial [105]. In this trial, 170 patients with MDS were randomized to receive either i.v. 5-AZA-CdR 15mg/m^2^ over three hours every eight hours for three consecutive days (135 mg/m^2^/course) every six weeks or the best supportive care (BSC). The overall response rate for the 5-AZA-CdR arm was 30%, compared with 7% for the BSC arm (*P* <0.001). The median duration of the response was 41 weeks with no difference in the time to AML progression compared with the BSC [105]. Similarly, the European Organization for Research and Treatment of Cancer and the German MDS Study Group conducted a large phase III multicenter trial that randomized 233 elderly patients with higher-risk MDS to receive either i.v. 5-AZA-CdR 15 mg/m^2^ over three hours every eight hours for three consecutive days (135mg/m^2^/course) every six weeks or the BSC [106]. This trial reported a 34% overall response rate with 5-AZA-CdR and an improvement in the progression-free survival (0.55 versus 0.25 years; *P* = 0.004) compared with the findings for the BSC group.

The time to AML progression and overall survival, however, did not differ significantly between the two groups. The main adverse effect due to 5-AZA-CdR treatment appears to be myelosuppression, including severe neutropenia, thrombocytopenia, and anemia. The incidence of myelosuppression, however, decreases in responding patients with the continuation of therapy. Grade 3/4 non-hematologic toxicity is rare and usually clinically insignificant.

MDS patients who fail 5-azacytidine treatment can respond to 5-AZA-CdR. In a study of 14 MDS patients after the failure of previously administered 5-azacytidine therapy, low-dose 5-AZA-CdR (20 mg/m^2^/day i.v. over 5 days) was investigated [107]. The overall response rate was 28% (four patients), including CRs in three patients.

In another study, the activity of 5-AZA-CdR was tested in 27 elderly patients with AML, MDS, or CML [108]. One CR was observed with the dose of 500 mg/m^2^/24 hours for the three-day schedule (estimated plasma drug concentration, 2 μM). There were five partial remissions. In this mostly ARA-C–resistant patient group, the efficacy of 5-AZA-CdR was unquestionable. Myelosuppression, however, was prolonged up to 42 days. At the time of this study, G-CSF injections were not available to accelerate bone marrow recovery.

In another study, the activity of 5-AZA-CdR was tested in 27 elderly patients with AML, MDS, or chronic myeloid leukemia (CML) [56]. 5-AZA-CdR was administered at doses of 30 to 90 mg/m^2^ for AML patients as a four-hour i.v. infusion three times a day for three days. The overall response rate of 45% was encouraging in this group of patients with refractory disease. On the basis of these promising outcomes, the same research group tested 5-AZA-CdR at higher doses in a cohort of 12 patients with AML [109]. The overall response rate was around 40% in this group of patients with poor prognoses. The promising results achieved in these two studies support the rationale for further investigations of 5-AZA-CdR in AML patients.

In a study of elderly AML patients, 5-AZA-CdR was administered at 20 mg/m^2^/day for five days every four weeks [110]. The overall response rate was around 26%. The median time to response was three cycles. Toxicities were similar to those in the previous studies at this dose level. In the study that led to the approval of 5-AZA-CdR for MDS by the FDA, a retrospective detailed investigation of bone marrow revealed that 12 patients with AML were misdiagnosed as MDS. Nine of these patients were randomly assigned to receive 5-AZA-CdR, and the response rate in this subgroup for 5-AZA-CdR was 56% [105]. Several studies have evaluated the combination of 5-AZA-CdR with other anti-cancer agents [111]. In this review, we have focused primarily on 5-AZA-CdR monotherapy.

Currently, there is no established care for AML patients who are not eligible to receive standard induction chemotherapy due to poor performance status. A phase III study that enrolled 485 patients who were at least 65 years of age with newly diagnosed, *de novo*, or secondary AML and intermediate or unfavorable risk cytogenetics was performed [112]. 5-AZA-CdR was given at a dose of 20 mg/m^2^/day as a one-hour infusion for five consecutive days every four weeks (estimated drug plasma concentration of 2 μM for one hour). Patients on 5-AZA-CdR had a median overall survival (OS) of 7.7 months, compared with 5 months in the control arm with a hazard ratio of 0.85. The stratified log-rank analysis, however, did not demonstrate a statistical significance between the groups. Subsequently, an unplanned OS analysis with one year of additional follow-up demonstrated the same improvement in median OS with a nominal *P* value of 0.037 (data on file). As of February 2012, the FDA concluded, based on statistical analysis, that 5-AZA-CdR does not appear to improve survival in older patients with AML [113]. The correlation of clinical responses and plasma levels of 5-AZA-CdR in hematologic malignancies and in MDS is summarized in Tables 6 and 7, respectively.

**Table 6:** Correlation of clinical responses and plasma concentrations of 5-AZA-CdR in patients with hematological malignancies

**Type of leukemia**	**Dose-schedule**	**Duration infusion**	**Estimated plasma concentration**	**Estimated AUC (μM × hour)**	**Response %ORR (%CR)**	**References**
AML^a^	*ind:*135 mg/m^2^*main*:20 mg/m^2^	1 to 72 hours	0.12 to 1.25 μM	0.12 to 90	26	[143]
AML	20 mg/m^2^	1 hour	1.25 μM	1.25	25 (24)	[110]
AML	20 mg/m^2^	1 hour	1.25 μM	1.25	64 (47)	[144]
CMML	15 mg/m^2^	3 to 4 hours	0.24 to 0.31 μM	0.72 to 1.24	25(11)	[105,145,146]
AML	20 mg/m^2^	1 hour	1.25 μM	1.25	67 (26)	[147]
CML	50 to 100 mg/m^2^	6 hour	0.52 to 1.0 μM	3.12 to 6.0	B28(10),A55(23),C63(13)	[148]
CML	50 to 100 mg/m^2^	6 hours	0.52 to 1.0 μM	3.12 to 6.0	53 (0)	[121,122]
CML	50 to 100 mg/m^2^	6 hours	0,52 to 1,0 μM	3.12 to 6.0	84 (10)	[121,122]
AML^b^	90 mg/m^2^	4 hours	1.41 μM	5.64	100 (100)	[149]
AML^c^	125 to 250 mg/m^2^	6 hours	1.3 to 2.6 μM	7.8 to 15.6	44 (44)	[150]
AML^d^	125 to 250 mg/m^2^	6 hours	1.3 to 2.6 μM	7.8 to 15.6	41 (41)	[151]
AML^d^	125 to 250 mg/m^2^	6 hours	1.3 to 2.6 μM	7.8 to 15.6	82(73)	[137]
AML	250 to 500 mg/m^2^	6 hours	2.61 to 5.2 μM	15.66 to 31.2	20 (0)	[137]
AML	270 to 360 mg/ m^2^/day t.i.d.	4 hours	4.2 to 5.6 μM	16.8 to 22.4	40 (30)	[109]
AML, ALL, CML	300 to 500 mg/m^2^/day c. i.v.	24 to 120 hours	0.16 to 1.3 μM	3.84 to 156.0	26 (4)	[108]
AML, ALL	37 to 80 mg/kg c.i.v.	36 to 60 hours	1.4 to 5.15 μM	50.4 to 309.0	37 (22)	[97]
AML, ALL	0.75 to 80 mg/kg conti.i.v.	12 to 44 hours	0.04 to 15 μM	9.0 to 660.0	14 (9)	[7]

^a^in combination with ATRA; ^b^in combination with daunorubicin; ^c^in combination with idarubicin; ^d^in combination with amsacrine. 1 μM= 228 ng/ml. 5-AZA-CdR, 5-aza-2′-deoxycytidine; A, accelerated; ALL, acute lymphocytic leukemia; AML, acute myeloid leukemia; ATRA, all trans retinoic acid; AUC, area under the curve; B, blastic; C, chronic; CML, chronic myeloid leukemia; CMML, chronic myelomonocytic leukemia; CR, complete remission; ORR, overall response rate.

**Table 7:** Correlation of clinical responses and plasma concentrations of 5-AZA-CdR in patients with MDS

**Dose-schedule**	**Duration of infusion**	**Estimated plasma concentration**	**Response % ORR (%CR)**	**References**
20 mg/m^2^/day i.v. q 5d	1hour	1.25 μM	34 (33)	[99]
10 mg/m^2^/day i.v. q 10d	1hour	0.63 μM		
15 mg/m^2^ i.v t.i.d. x 3d	4 hours	0.23 μM	20(15)	[123]
15 mg/m^2^ t.i.d. q3d	3 hours	0.31 μM	17 (9)	[105]

1 μM = 228 ng/ml. 5-AZA-CdR, 5-aza-2′-deoxycytidine; CR, complete remission; MDS, myelodysplastic syndrome; ORR, overall response rate.

### 5-Aza-CdR in the treatment of solid tumors

The anticancer activity of 5-AZA-CdR was studied in patients with refractory and metastatic solid tumors. In a phase I study of 5-AZA-CdR, the efficacy and toxicity of the drug were also explored in three children with metastatic solid tumor [7]. 5-AZA-CdR was given by continuous infusion. At doses in the range of 0.75 to 80 mg/kg for 12 to 44 hours, only limited activity was observed.

In a study of refractory metastatic prostate cancer patients, treatment with one-hour infusions of 75 mg/m^2^ 5-AZA-CdR, every eight hours for three doses stabilized the disease in 2 of 12 patients with an evaluable responses [114]. In another study, a seven-day continuous infusion of 5-AZA-CdR was investigated in patients with refractory solid tumors at a daily dose of 2 mg/m^2^[115]. The findings of this study demonstrated that genomic DNA methylation reverted to baseline levels by 28 to 35 days after the start of 5-AZA-CdR treatment. No objective responses were observed, however, probably due to the very low concentrations of 5-AZA-CdR in the body fluids. Only one patient with metastatic ovarian cancer and one patient with renal carcinoma had stable disease.

In a pilot phase I/II study performed in 15 patients with stage III/IV metastatic non-small cell lung cancer (NSCLC), 5-AZA-CdR was administered over eight hours as a continuous infusion at doses of 200 to 660 mg/m^2^[116]. The steady-state plasma level of 5-AZA-CdR was estimated to range from 1 to 5 μM. Three patients survived beyond 15 months, indicating that relatively high doses of 5-AZA-CdR had anti-tumor activity. One patient who received five cycles of 5-AZA-CdR survived for seven years. The hematopoietic toxicity of this schedule was acceptable. These interesting results warrant further investigation of 5-AZA-CdR for the treatment of NSCLC using longer durations of infusions (18 to 24 hours).

In summary, treatment of solid tumors with 5-AZA-CdR led to only limited responses. Notably, however, the blood concentration of 5-AZA-CdR and the exposure time to the drug in solid tumor studies were suboptimal when compared with the results achieved with higher concentrations in both *in vitro* and *in vivo* animal studies. As clearly demonstrated in the mouse model, doses of 5-AZA-CdR must be intensified to realize potent antitumor activity [60].

### Correlation of the plasma level of 5-AZA-CdR with chemotherapeutic action

*In vitro* colony-forming assays of leukemic cells revealed that there is a significant correlation among the concentration of 5-AZA-CdR, the exposure time, and its anticancer and demethylating effects [44,59]. A study on human myeloid, T-cell and B-cell lines, revealed that 10 μM 5-AZA-CdR for one hour produced a loss of clonogenicity of these cells lines that was 33%, 50%, and 49%, respectively. These observations confirm the S-phase specificity of 5-AZA-CdR, that is, only cells in S-phase, but not G1 and G2, are targets of this chemotherapy. During a one-hour treatment approximately 50% of the cells are present in S phase. With an exposure time of 24 hours, 1 μM 5-AZA-CdR produced 96%, >98%, and 100% losses of clonogenicity, respectively, for the three leukemic cell lines. These findings indicate the importance of the exposure time to 5-AZA-CdR, which should be long enough to permit the transit of all leukemic cells into S-phase. The doubling time of the cell lines was 20 to 24 hours [117].

The therapeutic and toxic effects of 5-AZA-CdR were investigated in mice with L1210 leukemia. The authors of one study concluded that a treatment duration of 24 hours, giving a 5-AZA-CdR plasma level of around 2 μM, would be most desirable for maximum cell kill [117]. In another study on L1210 leukemia, the curative dose of 5-AZA-CdR was estimated to be 20 mg/kg administered as an 18-hour infusion [60]. The estimated plasma concentration of 5-AZA-CdR for this infusion was approximately 3 μM.

The antileukemic activity of 5-AZA-CdR was also studied in the Brown Norway rat leukemia model, which is a good model of human AML [4]. A dose–response relationship was observed for 5-AZA-CdR for doses up to 50 mg/kg (administered every 12 hours × 3) in this rat model. This dose of 5-AZA-CdR produced a 100% increase in survival time. In this rat model of AML, 5-AZA-CdR was more effective than ARA-C (at a dose of 200 mg/kg per treatment). It is interesting that both 5-AZA-CdR and ARA-C reduced the number of normal hematopoietic stem cells in the bone marrow by 30% to 40% at a dose of 50 mg/kg, with no further reduction at higher doses (250 mg/kg). This is a clear indication that the resting normal hematopoietic stem cells survive intensive doses of S-phase–specific agents (administered over an interval of 24 hours) due to the presence of resting non-proliferating hematopoietic stem cells [4].

Studies of the antineoplastic action of 5-AZA-CdR in mouse models of solid tumors provide some insight into the importance of the dose schedule. The *in vivo/in vitro* EMT6 tumor model is an excellent tool to study the pharmacodynamics of antineoplastic agents [118]. In this model, EMT6 tumor cells from cell culture are injected subcutaneously (s.c.) into mice, and chemotherapy is administered when the tumor size is approximately 3 to 5 mm. After chemotherapy, the tumor is excised and trypsinized, and single-cell suspensions are plated in Petri dishes. Cell survival after chemotherapy is quantified by comparing the number of colonies to the number with controls. Using this assay, 5-AZA-CdR at a dose of 30 mg/kg as an 18-hour infusion markedly reduced the proliferative potential of the tumor stem cells [60]. The estimated plasma level of 5-AZA-CdR in this treatment was in the range of 4 μM. These preclinical results illustrate the importance of using intensive chemotherapy with 5-AZA-CdR to obtain a good antitumor response.

### Hematopoietic toxicity of 5-Aza-CdR

The major side effect of 5-AZA-CdR is myelosuppression. In patients with advanced hematologic malignancies, it is difficult to fully evaluate the hematopoietic toxicity of 5-AZA-CdR due to the presence of a large number of abnormal cells in the bone marrow, which can interfere with recovery after chemotherapy. Tumor patients without the presence of malignant cells in the bone marrow are good candidates for the evaluation of hematopoietic toxicity on ‘normal bone marrow’. Patients with lung cancer without prior chemotherapy treated with 660 mg/m^2^ 5-AZA-CdR as an eight-hour infusion exhibited significant recovery of the white blood cell count at approximately day 35 [116]. In patients with advanced acute leukemia, the recovery of the granulocyte count (>500/μl) after an intensive dose of 5-AZA-CdR occurred at about day 45 [97]. This observation suggests that the interval between cycles of 5-AZA-CdR therapy should be six weeks to permit adequate recovery of the granulocyte count. At low doses of 5-AZA-CdR (15 to 20 mg/m^2^), the drug is well tolerated in patients with MDS, and the primary hematological toxicity is transient neutropenia, which is predictable and manageable [92,111,119,120].

In a study of patients with CML, 5-AZA-CdR was administered at a dose of 500 to 1,000 mg/m^2^ over five days and prolonged neutropenia was the major side effect [121,122]. At doses of 75 to 100 mg/m^2^ delivered over six hours every 12 hours for 10 doses, the median time to neutrophil recovery above 500/μl was 50 (total dose of 1,000 mg/m^2^/cycle) or 45 days (total dose of 750 mg/m^2^/cycle), respectively.

## Discussion

5-AZA-CdR is an effective epigenetic drug for the treatment of hematologic malignancies. The clinical efficacy of 5-AZA-CdR is due to its demethylating epigenetic action, which reactivates tumor suppressor genes silenced by DNA methylation. Low-dose 5-AZA-CdR (20 mg/m^2^ one-hour infusion x five days or 15 mg/m^2^ four-hour infusion q8 h × three days) can produce CRs in patients with MDS and AML [99,123]. These low dose schedules cause less toxicity than intensive doses of 5-AZA-CdR. This is especially important for older patients with a poor performance status who are not good candidates for intensive therapy with 5-AZA-CdR. The interesting responses to low-dose 5-AZA-CdR suggest that leukemic stem cells are very sensitive to low concentrations of 5-AZA-CdR, an indication of the immense chemotherapeutic potential of this drug.

Recent preclinical reports indicate that very low dose 5-AZA-CdR administered frequently (two to three times/week) also has the potential to be an effective form of therapy for cancer. One reason for the failure of cytotoxic chemotherapy is that malignant cells can be resistant to the induction of apoptosis due to a non-functional p53 pathway as a result of mutations or deletions [124]. However, the genes that program terminal differentiation in these apoptosis-resistant malignant cells can be silenced by epigenetic mechanisms, such as DNA methylation, and reactivated by non-toxic doses of 5-AZA-CdR. This same treatment maintains the self-renewal of normal hematopoietic stem cells by preventing repression of stem cell genes by differentiation-inducing stimulus and induces differentiation of AML cells [54]. For these reasons the very low dose 5-AZA-CdR does not produce pronounced granulocytopenia as observed with intense doses of this agent.

Laboratory studies on AML cells support the use of very low-dose 5-AZA-CdR. 5-AZA-CdR inhibits *in vitro* proliferation, decreases colony formation and induces myeloid differentiation of p53-null AML cells [125]. These observations were confirmed using fresh AML cells from a patient. The AML cells were transplanted into NSG immunosuppressed mice and treated with a s.c. injection of 5-AZA-CdR (0.2 mg/kg three times/week for two weeks, then once/week). This very low dose 5-AZA-CdR was much more effective in prolongation of the survival time of the leukemic mice than an intense dose of ARA-C (75 mg/kg per day intraperitoneally for five days). The proof of principle of the very low-dose 5-AZA-CdR was also confirmed in a clinical trial in MDS patients with high-risk cytogenetics [126]. 5-AZA-CdR 3.5 to 7 mg/m^2^ administered one to three times/week produced an overall response of 84% (CR + hematologic improvement + stable disease), which is remarkable. Complete cytogenetic remissions were observed in 50% of the patients. It will be interesting to see if this non-toxic differentiation therapy with 5-AZA-CdR will be effective in older AML patients who are not candidates for cytotoxic chemotherapy.

One limitation of the low dose 5-AZA-CdR for the treatment of AML or MDS is the problem of eradicating malignant cells in the liver or spleen due to the high activity of cytidine deaminase. Deamination of 5-AZA-CdR can reduce its concentration to sub-therapeutic levels in these organs. The use of an inhibitor of cytidine deaminase, such as tetrahydrouridine, in combination with 5-AZA-CdR has the potential to overcome this problem [127]. The proof of principle of this approach was demonstrated in a murine xenotransplant model of AML where tetrahydrouridine produced a marked enhancement of the antineoplastic activity of 5-AZA-CdR [128]. The combination of these agents merits a high priority for clinical investigation in patients with hematologic malignancies.

Preclinical studies indicate that very low-dose 5-AZA-CdR also has the potential to be an effective treatment for tumors with a favorable epigenetic signature. As an example, human renal carcinoma cells derived from a patient were inoculated s.c. into nude mice followed by treatment with low dose 5-AZA-CdR (0.2 mg/kg s.c. × 3/week) [129]. This low dose therapy was very effective in reducing tumor growth and did not produce leukopenia. This low dose 5-AZA-CdR, in combination with tetrahydrouridine to inhibit cytidine deaminase, was also very effective in inhibiting the growth of murine melanoma tumors in mice [130]. The low dose chemotherapy did not produce leukopenia or reduction in body weight. 5-AZA-CdR was also shown to induce differentiation of both human and murine melanoma cell lines.

These observations on very low 5-AZA-CdR therapy of leukemia and tumors were confirmed by Tsai *et al*. [131] using a different dose-schedule. These investigators showed that low dose 5-AZA-CdR (72-hour exposure) reduced colony formation of AML cells from patients, but not the normal hematopoietic stem cells colony-forming units-granulocyte macrophage (CFU-GM). The low dose 5-AZA-CdR (0.1 μM, 72 hours *ex vivo*) followed by 7 to 14 days drug-free media was also shown to decrease tumorigenicity in mouse tumor xenografts.

In summary, the very low dose 5-AZA-CdR preclinical studies showed that this type of treatment could produce a loss in the self-renewal potential of cancer stem cells due to the increase in the expression of genes that suppress malignancy. These epigenetic changes are maintained in the target cells after drug removal and accumulate with each low dose treatment until there is a complete loss of cancer stem cell potential. The low dose chemotherapy merits clinical investigation in patients with cancer. The very low dose 5-AZA-CdR may also have the potential to maintain CR in patients with leukemia and arrest malignant progression in patients with solid tumors. For cancer patients with poor performance status the very low dose 5-AZA-CdR therapy may be a good option to improve the quality of life rather than the use of only supportive therapy or no treatment.

Some patients with cancer may not respond or show disease progression on the low dose-schedule 5-AZA-CdR. This may be due to fact that: a) the cancer may have an epigenetic/genetic signature that is not predisposed to the induction of terminal differentiation by low dose 5-AZA-CdR; b) the target cancer cells may have a low level of deoxycytidine kinase, the enzyme that activates the prodrug, 5-AZA-CdR [132]; c) the cancer cells may be in anatomic sanctuaries that have low penetration of 5-AZA-CdR (for example, CSF, testis, tumors with a limited blood supply [133]). The concentration of 5-AZA-CdR in these sanctuaries is too low to eliminate the cancer stem cells; d) the cancer cells may be in a biochemical sanctuary that contains high levels of cytidine deaminase (for example, liver, spleen); e) drug resistance develops more rapidly after repetitive treatments with low-dose chemotherapy; f) because 5-AZA-CdR is a cell cycle-specific agent, a one- to four-hour infusion of this agent only targets cancer cells in S-phase, whereas cells in G1 and G2 phases escape the chemotherapeutic action of this analog during short-term treatment. A long interval (12 to 24 hours) between infusions can also permit leukemic stem cells to pass through the S-phase cell cycle without exposure to 5-AZA-CdR and, thus, escape its antileukemic action. This possibility was demonstrated in a preclinical study on leukemia using the S phase–specific drug ARA-C [134].

One approach to overcome these caveats is to use intensive chemotherapy with 5-AZA-CdR administered as a continuous infusion for patients with leukemia. This objective is of high priority and involves determination of the optimal plasma level of 5-AZA-CdR and duration of treatment that can eliminate leukemic stem cells in these sanctuaries. The *in vitro* data on colony assays of human leukemic cell lines indicate that a concentration of 5-AZA-CdR in the range of 1 to 2 μM for the duration of the cell cycle of the leukemic cells has the ability to completely eliminate their proliferative potential. Another approach is to use 5-AZA-CdR in combination with an inhibitor of cytidine deaminase, such as tetrahydrouridine or zebularine, to target the leukemic cells in the biochemical sanctuaries [135,136]. An additional approach is to use 5-AZA-CdR in combination with a biochemical modulator, such as 3-deazauridine, to eliminate drug-resistant leukemic cells due to a deficiency in deoxycytidine kinase [132]. Cancer cells deficient in deoxycytidine kinase are very sensitive to the cytotoxic action of 3-deazauridine [132].

One of the key points concerning intensive-dose therapy with 5-AZA-CdR is the fact that it produces delayed and prolonged myelosuppression. Several investigators have used intensive-dose 5-AZA-CdR in patients with advanced leukemia and observed that most of these patients with a good performance status recovered from the hematopoietic toxicity. These early studies were performed before the clinical use of granulocyte colony stimulating factors to accelerate the recovery from myelosuppression. Some examples of the dose schedules of intensive-dose 5-AZA-CdR that were used are: total dose of 2,479 mg/m^2^ as a 60-hour i.v. infusion; 500 mg/m^2^ as a 6-hour infusion every 12 hours × 5 days for a total dose of 5,000 mg/m^2^; and a daily dose of 300 to 500 mg/m^2^ as a 24 to 120 hour infusion [97,108,137]. The estimated plasma level of 5-AZA-CdR in these studies ranged from 1 to 3 μM. The hematopoietic toxicity produced by intensive doses of 5-Aza-CdR can also be predicted from its comparative pharmacology with the related deoxycytidine analogue, ARA-C. Both 5-AZA-CdR and ARA-C are S-phase–specific agents. They have identical metabolism, and their antineoplastic action is due to their incorporation into DNA, but their molecular mechanisms of action differ. 5-AZA-CdR inhibits DNA methylation, whereas ARA-C potently inhibits DNA replication. Because they target the same cells (proliferating cells in S phase), they should produce a similar pattern of hematopoietic toxicity. Most leukemic patients in CR with a good performance status recover from the hematopoietic toxicity produced by very high-dose ARA-C (up to 6,000 mg/m^2^/day for four days; total dose 24,000 mg/m^2^) [138]. These observations provide a rationale for intensive doses of 5-AZA-CdR in the range of 1,000 mg/m^2^/day in leukemic patients in CR without encountering unacceptable hematologic toxicity for patients with a good performance status. The recovery from granulocytopenia after 5-AZA-CdR is approximately two weeks longer than that after ARA-C [97]. This is probably due to the delayed epigenetic action of 5-AZA-CdR on normal hematopoietic stem cells compared with the acute cell kill produced by ARA-C. From this point of view, it is better to use a six-week interval between cycles of 5-AZA-CdR rather than the four-week interval used for ARA-C.

It is a remarkable achievement that current chemotherapy can induce CR in most patients with hematologic malignancies. The major challenge is maintaining the patients in CR. Patients in CR are good candidates for experimental chemotherapy because of their good hematologic status. From an ethical point of view, high-risk leukemic patients with an unfavorable karyotype that predicts a poor outcome are good candidates for intensive therapy with 5-AZA-CdR.

Pharmacokinetic/pharmacodynamic calculations can be used to estimate the optimal dose for 5-AZA-CdR. For the initial studies, we recommend a combination of intensive and low-dose 5-Aza-CdR to treat high-risk patients with leukemia. For the initial intensive phase, 5-AZA-CdR can be infused at a rate of 30 mg/m^2^/hour for days one and two (total dose 1,440 mg/m^2^/day). This infusion rate should give a plasma concentration of approximately 2 μM, shown to be very effective in both *in vitro* and *in vivo* animal studies on leukemia. The objective of this intensive therapy of 5-AZA-CdR is to target the most rapidly proliferating leukemic stem cells and those in anatomic and biochemical sanctuaries. The intensive phase is followed by a low-dose phase where 5-AZA-CdR is administered on days three and four as a short infusion at a dose of 30 mg/m^2^/day. The objective of this phase is to target cancer stem cells with a long cell cycle that do not enter the S phase during the first two days of treatment. Supportive care with granulocyte colony stimulating factor is recommended to shorten the duration of granulocytopenia. The interval between each cycle of therapy should be six weeks to permit adequate recovery from bone marrow toxicity. Modifications of the proposed dose schedule may be required for optimization. In subsequent studies, the low-dose phase of 5-AZA-CdR can be replaced by histone deacetylase (HDAC) and/or histone methylation inhibitors, which showed a synergistic interaction against leukemic cells in preclinical studies [139,140]. It could also be interesting to design a clinical study in AML patients to see the efficacy and safety of decitabine in combination with promising novel tyrosine kinase inhibitors such as quizartinib [141]. Both preclinical and clinical observations indicate that 5-AZA-CdR has tremendous potential for the treatment of hematologic malignancies. The results of this proposed clinical trial on 5-AZA-CdR will be of great interest and will hopefully lead to improved overall survival of patients with advanced leukemia.

Chemotherapy of solid tumors using 5-AZA-CdR also merits clinical investigation. Most malignancies have a large number of tumor suppressor genes that are silenced by aberrant DNA methylation, providing many interesting targets for 5-AZA-CdR therapy. The preclinical data indicate that all types of tumors are sensitive to 5-AZA-CdR treatment, including low dose therapy. However, the clinical responses to low-dose 5AZA-CdR in solid tumors are reported to be very limited. It should be noted, however, that the estimated plasma levels of 5-AZA-CdR in these trials was too low. *In vitro* clonogenic assays using an exposure time of 24 hours on tumor cells indicate that low concentrations of 5-AZA-CdR are not very effective. Our preclinical data on chemotherapy of tumors in the mouse model suggest that the plasma level of 5-AZA-CdR should be approximately 3 μM for curative therapy [60].

An example of the potential of intensive doses for tumor therapy is the pilot stage III/IV NSCLC study where the patients were administered 5-AZA-CdR (660 mg/m^2^ as an eight-hour infusion), which produced a plasma concentration in the range of 3 μM. This study produced some promising results: three patients who survived for 15 months and one patient who survived for seven years [116]. The preclinical data support the possibility of this type of response and predict that an infusion time longer than eight hours should be more effective. One major reason for the failure of tumor chemotherapy is the limited penetration of drugs into tumors [133]. One approach to overcome this problem is to obtain high plasma concentrations of anticancer drugs to enhance their penetration into tumors. This provides a rationale for the use of intensive-dose 5-AZA-CdR for the treatment of solid tumors. S phase–specific drugs can be used at very high doses for a limited duration without unacceptable side effects. Patients with metastatic malignancy and poor prognosis are potentially good candidates for intense chemotherapy with 5-AZA-CdR. As an initial study, we suggest a dose schedule of 60 mg/m^2^/hour administered as an 18-hour infusion (total dose of 1,080 mg/m^2^). This dose schedule will give an estimated steady state plasma concentration in the range of 4 μM. The interval between cycles should be six weeks to permit adequate bone marrow recovery*.* Patients treated previously with intensive cytotoxic chemotherapy are at risk of severe hematopoietic toxicity and require a minimum of four weeks of recovery before being eligible for intensive 5-AZA-CdR therapy. Patients who do not respond to anticancer agents that do not produce hematopoietic toxicity would also be good candidates for this investigational therapy. Depending on the response, the intensive dose schedules for 5-AZA-CdR may have to be modified for optimization (for example, increase the dose and/or duration of the infusion; use low dose 5-AZA-CdR between cycles of intensive doses). The goal should be to optimize the dose-schedule of 5-AZA-CdR to reveal its potential for tumor therapy. It may also be possible to increase the effectiveness of this tumor therapy by using a sequential treatment of 5-AZA-CdR followed by an inhibitor of histone modification [139,140] or with a tyrosine kinase inhibitor [142].

## Conclusion

Epigenetic changes, such as DNA methylation, play a very important role in both leukemogenesis and tumorigenesis. Because these epigenetic changes are very prominent in cancer cells and are reversible, they are potential targets for chemotherapy with 5-AZA-CdR. Pharmacodynamic and pharmacokinetic analysis of 5-AZA-CdR from both preclinical and clinical data can provide insight toward optimizing cancer treatment using this interesting epigenetic agent.

## Abbreviations

5-AZA-CdR: 5-Aza-2′-deoxycytidine; ALL: Acute lymphocytic leukemia; AML: Acute myeloid leukemia; ARA-C: Cytosine arabinoside; BSC: Best supportive care; CFU-S: Colony forming unit in spleen; CML: Chronic myeloid leukemia; CMML: Chronic myelomonocytic leukemia; CpG: Cytosine-phosphate-guanine; CR: Complete remission; CSF: Cerebrospinal fluid; D50: 50% decomposition rates; DGU: *N*′-β-d-2-deoxyribofuranosyl-3-guanylurea; DNMT: DNA methyltransferase; FDA: US Food and Drug Administration; G-CSF: Granulocyte colony stimulating factor; HDAC: Histone deacetylase; HPLC: High performance liquid chromatography; HPLC/MS: High performance liquid chromatography/mass spectrometry; IC50: Half maximal inhibitory concentration; i.p: Intraperitoneal; MDS: Myelodysplastic syndrome; MTD: Maximally tolerated dose; NFDU: *N*-(formylamidino)-*N*′-β-d-2-deoxyribofuranosylurea; NSCLC: Non-small cell lung cancer; OS: Overall survival; s.c: Subcutaneous.

## Competing interests

The authors declare that they have no competing interests.

## Authors’ contributions

All authors contributed to the content. All authors read and approved the final manuscript.
